# Fracture Strength of Endocrowns Fabricated From Three Different Computer-Aided Design/Computer-Aided Manufacturing Ceramic Materials: An In-Vitro Study

**DOI:** 10.7759/cureus.41531

**Published:** 2023-07-07

**Authors:** Shatha Alshali, Esra Attar

**Affiliations:** 1 Department of Oral and Maxillofacial Prosthodontics, King Abdulaziz University, Jeddah, SAU

**Keywords:** vita suprinity, vita enamic, ips e.max cad, fracture strength, endocrowns

## Abstract

Introduction: Endocrown restorations have increased in popularity in recent years due to the advancement of both adhesive and restorative materials. The clinical success of endocrowns depends on several factors, including preparation design, material selection, fracture resistance, and marginal adaptation. The aim of this in vitro study was to compare the fracture strength of endocrown restorations fabricated from three different computer-aided design (CAD)/computer-aided manufacturing (CAM) materials.

Methods: Thirty extracted mandibular first molars were selected. The teeth underwent conventional root canal treatment before being prepared for endocrown restoration. The teeth were allocated to three groups (*n*=10), corresponding to each of the three ceramic materials used to fabricate the endocrowns they would receive. The ceramic materials used were zirconia-reinforced lithium silicate glass-ceramic (VITA Suprinity, VITA Zahnfabrik, Bad Säckingen, Germany), polymer-infiltrated hybrid ceramic (VITA Enamic, VITA Zahnfabrik, Bad Säckingen, Germany), and lithium disilicate glass-ceramic (IPS e.max CAD, Ivoclar Vivadent, Schaan, Liechtenstein). The specimens were scanned, and the digital impressions were transferred into design software to construct the endocrowns. The endocrowns were then milled and cemented. A universal testing machine (5969L3504, Instron, USA) was used for the fracture strength test at a crosshead speed of 1 mm/min until catastrophic failure occurred. Statistical analysis was performed using IBM Corp. Released 2015. IBM SPSS Statistics for Windows, Version 23.0. Armonk, NY: IBM Corp.

Results: The one-way analysis of the variance test indicated a significant difference in the fracture strength between the different ceramic groups tested (*P*=0.037). The Tukey posthoc test showed that the IPS e.max CAD group had marginally higher fracture strength values than the VITA Enamic group (*P*=055). No significant differences in fracture strength values were found between the VITA Enamic and VITA Suprinity groups or between the VITA Suprinity and IPS e.max CAD groups (*P*>0.05).

Conclusion: The reported fracture strength values for all the tested materials were higher than the strength required to resist masticatory forces. Therefore, endocrowns fabricated using VITA Enamic, IPS e.max CAD, and VITA Suprinity CAD/CAM materials present restorations with a clinically acceptable fracture strength.

## Introduction

The restoration of badly damaged teeth has always presented a challenge. In addition to the coronal destruction, the loss of strength of endodontically treated teeth is attributed to the removal of dentin during the pulpectomy and the instrumentation associated with root canal treatment. When the coronal retention of the restoration is compromised, posts and cores have been conventionally utilized, but they require further removal of the intraradicular dentin. Therefore, other restorative approaches have been introduced, including endocrown restorations [1]. Pissis was the first to describe this type of restoration in 1995 [2], while Bindl and Mormann introduced the term “endocrown” in 1999 [3]. An endocrown is a monoblock ceramic crown that utilizes the internal portion of the pulp chamber along with the cavity margins and the adhesive cementation for retention [4,5].

The advantages of endocrown restorations over conventional post/core restorations include preservation of the tooth structure and reduced chair-side time [1]. Previous investigations reported that the fracture strength of molars restored with endocrowns was superior to that of those restored with post/core-retained crowns [6], while Huda et al. reported that the fracture strength of teeth restored with endocrowns was higher than that of those restored with inlay and onlay restorations [7].

The clinical success of an endocrown depends on several factors, including preparation design, material selection, fracture resistance, and marginal adaptation [8,9]. These restorations have increased in popularity in recent years due to the advancement of both adhesive and restorative materials [10,11].

Ceramic or resin-based materials can be used in the fabrication of endocrowns using either milling or pressing techniques [8,12]. Lithium disilicate glass ceramics, zirconia-reinforced glass ceramics, and resin-infiltrated ceramics have been manufactured with various esthetic and mechanical properties to enhance both clinical performance and patient satisfaction [8,9]. Lithium disilicate glass ceramics have increased in popularity in the area of endocrown fabrication due to their superior mechanical and esthetic properties, their ability to etch and bond to the tooth structure, and their enhanced durability [9,13,14].

Resin-based or polymer-infiltrated computer-aided design (CAD)/computer-aided manufacturing (CAM) ceramic blocks were designed to combine the mechanical properties of resin and ceramic materials. They have been recommended for use in place of conventional ceramics since their moduli of elasticity are closer to dentin [8,9,15]. They can absorb the masticatory stresses and can be placed as bulk bases in high-stress-bearing areas [15]. Acar et al. reported that hybrid blocks possess adequate fracture strength for utilization in the fabrication of endocrowns [16]. Resin-based ceramics offer less crack propagation and provide higher fracture resistance than some CAD/CAM ceramics [14]. When the modulus of elasticity differs considerably between the dentin and the ceramic material, this can result in debonding or even fracture of the tooth, the ceramic material, or both. Hence, the material properties and fabrication technique are of critical importance to superior clinical performance [8]. Some authors have limited the indication of endocrowns to teeth with short clinical crowns or cases with reduced vertical dimensions, anticipating that more flexing can be expected with an increased endocrown height, which can lead to debonding of the restoration or tooth fracture [13].

Recently, silicate-based ceramics have been reinforced with zirconia to improve their deformation resistance. Zirconia-reinforced silicate ceramics may reduce the mechanical failure of root canal-treated teeth restored with adhesive restorations, especially in cases of increased functional activity [17].

This in-vitro study aimed to compare the fracture strength of endocrowns milled of three CAD/CAM ceramics: polymer-infiltrated hybrid ceramic (VITA Enamic, VITA Zahnfabrik, Bad Säckingen, Germany), zirconia-reinforced lithium silicate glass ceramic (VITA Suprinity, VITA Zahnfabrik, Bad Säckingen, Germany), and lithium disilicate glass-ceramic (IPS e.max CAD, Ivoclar Vivadent, Schaan, Liechtenstein).

## Materials and methods

This in vitro study was conducted at King Abdulaziz University (KAU), Jeddah, Saudi Arabia. The study was approved by the Research Ethics Committee of the Faculty of Dentistry at KAU. The sample allocation for this study and the specimen preparation discussed in this section are as described by the authors in a previous investigation [18]. Thirty extracted mandibular first molar teeth were inspected for cracks, caries, and restorations before being cleaned ultrasonically and stored in a saline solution. Specimens were then mounted in autopolymerizing acrylic resin (Jet XR, Lang Dental, Wheeling, IL) using customized molds. The acrylic resin extended 2 mm below the CEJ. Root canal treatment was performed as follows:

Access preparation and working length determination: Access to the root canal system was achieved using a #4 or a #6 high-speed long shank round bur under copious water flow. De-roofing was achieved using either a round or an Endo Z bur. The canals were negotiated and explored using size-10 K-files (Dentsply Sirona, Charlotte, North Carolina, USA).

Biomechanical instrumentation: After determining the working length, establishing a coronal flare, and attaining a glide path using size #10 or #15 hand files, instrumentation of the canals was done using NiTi-rotary files (ProTaper Next, Dentsply Sirona, Charlotte, North Carolina, USA) up to size 30/07. The pulp floor was filled with 4% sodium hypochlorite (NaOCl) throughout the instrumentation and regularly replaced after each file. A 10-ml syringe was used with a 30-gauge needle placed 1 mm away from the established length. Sterile paper points were used to dry the canals.

Obturation: Continuous wave compaction obturation method was followed. A resin-based sealer, AH Plus, was introduced to the canals using Lentulo spirals. A matched gutta-percha cone (ProTaper Next Conform Fit, Dentsply Sirona, Charlotte, North Carolina, USA) was selected. A hot condenser B&L alpha unit was used to down pack the gutta-percha, and a B&L Beta device was used to backfill the canal (B&L Biotech USA, Inc., Fairfax, Virginia, USA).

Teeth preparation: Tooth preparation for the endocrown restorations was performed as follows: Occlusal reduction was carried out using a wheel diamond bur held parallel to the occlusal plane, 2 mm above the cementoenamel junction, to prepare the cervical margin. A shoulder finish line was prepared with a width of 1.5-2 mm. Axial preparation was performed to eliminate any undercuts from the access cavity using a round-end tapered diamond bur. The occlusal convergence was between 7° and 10°. All internal line angles were rounded. A flowable composite (3M TM Filtek Supreme Flowable Restorative. 3M, St. Paul, MN, USA) was used to create a flat base for the access cavity. The depth from the base of the pulp chamber to the prepared occlusal cervical margin was 3-5 mm, with the depth verified using a graded periodontal probe.

The teeth were allocated to three groups (n=10), corresponding to each of the three ceramic materials used to fabricate the endocrowns they will receive (Table 1).

**Table 1:** The ceramic materials, their composition, and their manufacturer

Material	Composition	Manufacturer
IPS e.max CAD	Lithium disilicate (Li_2_Si_2_O_5_)-based ceramic containing 70% Li_2_Si_2_O_5 _crystals	Ivoclar Vivadent, Schaan, Liechtenstein
VITA Suprinity	Zirconia-reinforced lithium silicate ceramic containing Li_2_Si_2_O_5 _crystals and 10% ZrO_2_	VITA Zahnfabric, Bad Säckingen, Germany
VITA Enamic	Polymer-infiltrated feldspathic ceramic (86% ceramic network and 14% acrylate polymer)	VITA Zahnfabric, Bad Säckingen, Germany

Endocrown fabrication: The specimens were scanned using a dental laboratory CAD/CAM scanner (i3Dscan, imes-icore, Eiterfeld, Germany). The scans were saved as standard tessellation language files and subsequently transferred into CORiTEC design software (CORiTEC SmartControl, imes-icore, Eiterfeld, Germany) to construct the corresponding endocrowns. Ceramic blocks were milled using a CORiTEC 250i Loader Pro system (imes-icore, Eiterfeld, Germany). IPS e.max CAD samples were crystallized using a ceramic furnace (Programat EP 5000, Ivoclar Vivadent, Schaan, Liechtenstein) for 30 min at a temperature of 840℃-850℃. VITA Suprinity samples were crystallized in the ceramic furnace for 26 min in accordance with the manufacturer’s instructions. All endocrowns were then glazed and polished.

**Figure 1:**
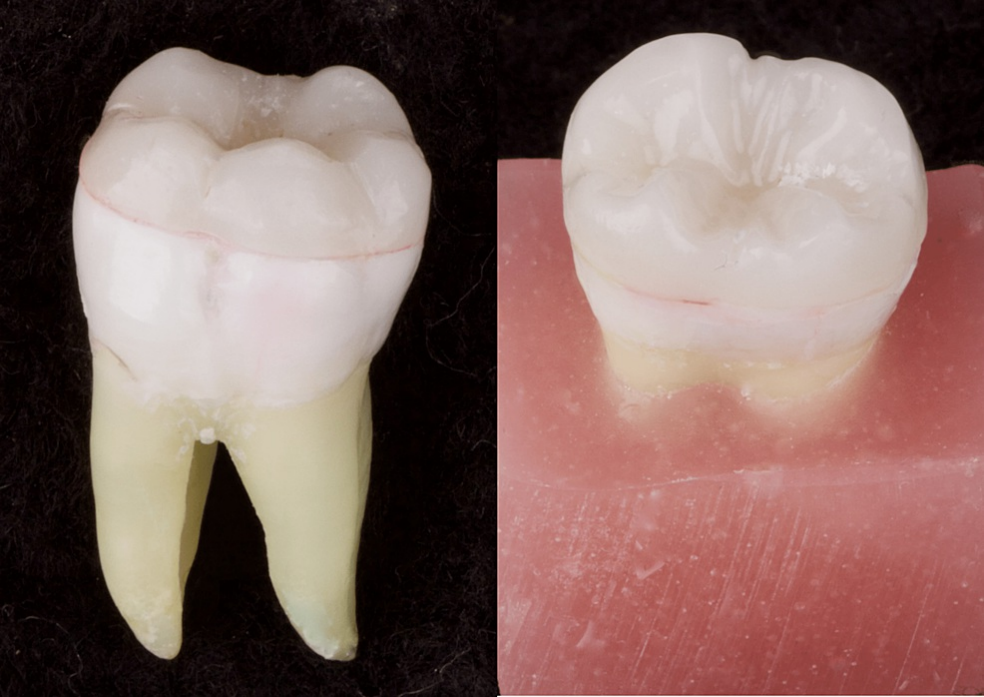
Specimen of the prepared tooth and corresponding milled endocrown

Cementation: The prepared surfaces of the teeth were etched with 35% phosphoric acid (N-etch, Ivoclar Vivadent, Schaan, Liechtenstein) for 15 s before being rinsed and dried. The internal surface of the endocrowns was etched using 5% hydrofluoric acid (HF5% Vita Ceramic Etch, VITA Zahnfabric, Bad Säckingen, Germany) for 20 s and then rinsed and dried. A silane coupling agent (Monobond Plus, Ivoclar Vivadent, Schaan, Liechtenstein) was applied for 60 s and then air dried. Dual-cure resin cement (3m ESPE Relyx U200 self-adhesive resin cement) was used as a luting agent to adhesively bond the endocrowns to their corresponding prepared teeth. Seating of the endocrowns was performed using a Multitest 2.5i machine under a 5-kg static load. Light curing was performed for at least 20 s per surface, and any excess resin cement was removed. The specimens were kept in saline following the process (Figure 1).

Fracture strength testing: A universal testing machine (5969L3504, Instron, USA) was used for the fracture strength test. The load was applied parallel to the long axis of the tooth on the center of the occlusal surface to simulate the loading conditions of the masticatory system in the molar area. A load was applied using a stainless-steel piston with a 0.5-mm radius at a cross-head speed of 1 mm/min until catastrophic failure occurred. Each specimen was positioned and fixed to maintain an identical loading position. The maximum force required to produce a fracture was recorded in Newtons (N).

Statistical analysis was performed using IBM Corp. Released 2015. IBM SPSS Statistics for Windows, Version 23.0. Armonk, NY: IBM Corp. Descriptive statistics were calculated for each group in terms of means and standard deviations. A one-way analysis of variance (ANOVA) was performed to compare the difference in fracture strength between the three ceramic groups. A Tukey post-hoc test was also conducted to ascertain which specific pairs of means were significantly different. All statistical tests were conducted at a significance level of P<0.05.

## Results

Descriptive statistics, in terms of means of the fracture strength values, standard deviations, and confidence intervals, are presented in Table 2.

**Table 2:** Mean values, standard deviations, and confidence intervals for the fracture strength of the three tested groups

	n	Mean (n)	Std. Deviation	Minimum (n)	Maximum (n)	
						VITA Enamic	10	1,232.1	137.3	1,045.0	1,423.5	
VITA Suprinity	10	1,488.4	253.0	1,251.2	1,997.9	
IPS e.max CAD	10	1,505.5	325.4	1,113.0	1,995.5	
Total	30	1,408.7	273.4	1,045.0	1,997.9	

Table 3 describes the one-way ANOVA test results. A significant difference in the fracture strength between the different ceramic groups tested (P=0.037) was noted. Therefore, the material had a significant effect on the fracture strength. 

**Table 3:** One-way analysis of variance (ANOVA) group comparison of the mean fracture strength values of the tested ceramic materials

	Sum of Squares	df	Mean Square	F	Sig.
Between Groups	469,198.1	2	234,599.0	3.728	.037
Within Groups	1,699,111.2	27	62,930.0		
Total	2,168,309.2	29			

The Tukey HSD post-hoc test results for paired groups revealed that the IPS e.max CAD group had marginally higher fracture strength values than the VITA Enamic group (P=055). However, no significant differences in fracture strength values were found between the VITA Enamic and VITA Suprinity groups or between the VITA Suprinity and IPS e.max CAD groups (P>0.05) (Table 4).

**Table 4:** Post-hoc multiple comparisons (Tukey HSD test) of the fracture strength for the tested ceramic materials

(I) material	(J) material	Mean Difference (I–J)	Std. Error	Sig.	95% Confidence Interval	
					Lower Bound	Upper Bound
VITA Enamic	VITA Suprinity	−256.3	112.2	.075	−534.5	21.8
	IPS e.max CAD	−273.4	112.2	.055	−551.6	4.7
VITA Suprinity	VITA Enamic	256.3	112.2	.075	−21.8	534.5
	IPS e.max CAD	−17.1	112.2	.987	−295.3	261.1
IPS e.max CAD	VITA Enamic	273.4	112.2	.055	−4.7	551.6
	VITA Suprinity	17.1	112.2	.987	−261.1	295.3

## Discussion

This study investigated the fracture strength of endocrowns fabricated with different CAD/CAM ceramics, namely, IPS e.max CAD, VITA Enamic, and VITA Suprinity. The results revealed that there was a significant difference in the fracture strength between the different ceramic groups tested.

Lithium disilicate glass ceramics are low-glass-content, particle-filled glass ceramics. The material contains 70% lithium disilicate crystals that enhance the mechanical properties and resistance to crack propagation. The CAD/CAM blocks (e.g., IPS e.max CAD) are supplied in a pre-crystallized (blue) state and exhibit a low flexural strength ranging between 30 and 130 MPa. Firing after milling is necessary to achieve final crystallization, which will achieve the final superior properties of the material, both mechanically and esthetically [19].

To enhance the mechanical properties of lithium silicate ceramics, manufacturers introduced zirconia-reinforced lithium silicate glass-ceramic materials, including VITA Suprinity, a lithium disilicate ceramic with 10% zirconia. Zirconium particles increase strength and enhance deformation resistance [17]. Elsaka et al. reported that zirconia-reinforced lithium disilicate ceramics have a higher flexural strength and fracture toughness than lithium disilicate ceramics alone [20]. The CAD/CAM blocks are provided in a pre-crystallized stage, and after firing, they reach their fully crystallized form [20,21]. Ceramic materials reinforced with polymers were introduced to combine the mechanical properties of ceramics and polymers. Importantly, these materials have a more comparable modulus of elasticity to dentin. They are typically composed of 86% ceramic and 14% polymer [9].

Gresnigt et al. compared the fracture resistance of endocrowns under axial and lateral forces and reported values of 2,428 ± 566 N for LAVA Ultimate CAD and 2,675 ± 588 for IPS e.max CAD under axial loading, while under lateral loading, the IPS e.max CAD exhibited significantly higher values [4]. Another study tested the fracture resistance of teeth restored with e.max endocrowns and concluded that the fracture resistance values under axial loading were far above the average human masticatory force, which is known to be 600-900 N [13]. The present study reported a higher fracture resistance of the endocrowns fabricated using IPS e.max CAD, VITA Enamic, and VITA Suprinity than that required under average masticatory forces. Saglam et al. reported that IPS e.max CAD had significantly higher fracture strength values (714.83 ± 150.73 N) than VITA Suprinity and VITA Enamic (569.39 ± 103.21 and 578.81 ± 150.73 N, respectively) [8]. The results of the present study were in accordance with Saglam et al.’s, with the fracture strength values found to be 1,505 ± 325, 1,488 ± 253, and 1,232 ± 137 N for IPS e.max CAD, VITA Suprinity, and VITA Enamic, respectively. Frankenberger reported the fracture strength values for IPS e.max CAD at between 723 and 736 N and between 702 and 733 N for Celtra Due, a zirconia-reinforced lithium silicate ceramic (Celtra Due, Dentsply Sirona, Konstanz, Germany) [22]. A study by Aktas et al. concluded that the modification of silica-based ceramics with either zirconia or polymer does not significantly affect the fracture strength of endocrowns, with the values reported as 1,085.33 and 1,025 N for Suprinity and Enamic, respectively [17]. The results of the present study are in line with the above two studies, with no significant difference found between VITA Suprinity and IPS e.max CAD or between VITA Enamic and VITA Suprinity.

To ensure standardization, all cavity preparations were carried out by the same operator. Variability in the endocrown dimension was minimized using a digital scanner and design software to construct the corresponding endocrowns. Ceramic blocks were milled under wet processing using a CORiTEC 250i Loader Pro system (imes-icore, Eiterfeld, Germany). All of this allowed for the standardization of the restoration in terms of occlusal morphology and cuspal inclination and hence, the standardization of the area and direction of the load application during testing. The seating of the endocrowns was performed using a Multitest 2.5i machine under a 5-kg static load. To mimic intraoral conditions, the loading direction for examining the fracture strength was applied parallel to the long axis of the tooth to simulate masticatory forces in the molar region.

The limitations of this study include that the load was applied axially only, which means the clinical scenarios where the teeth are subjected to off-axis, repetitive, cyclic fatigue masticatory stresses were not represented. Further investigations are needed to evaluate the modes of failure associated with the use of different ceramics.

## Conclusions

The findings of this study revealed that the fracture strength of IPS e.max CAD is significantly higher than that of VITA Enamic. However, the reported fracture strength values for all the tested materials were higher than the strength required to resist masticatory forces. Therefore, endocrowns fabricated using VITA Enamic, IPS e.max CAD, and VITA Suprinity CAD/CAM materials present restorations with a clinically acceptable fracture strength.
